# Analyzing the farmers’ pro-environmental behavior intention and their rural tourism livelihood in tourist village where its ecological environment is polluted

**DOI:** 10.1371/journal.pone.0247407

**Published:** 2021-03-11

**Authors:** Hongjiu Tang, Zhaoyin Liu, Xiaojie Long

**Affiliations:** 1 College of Landscape Architecture and Art Design, Hunan Agricultural University, Changsha, China; 2 School of Geography and Planning, Sun Yat-Sen University, Guangzhou, China; 3 College of Resources and Environment, Hunan Agricultural University, Changsha, China; Institute for Advanced Sustainability Studies, GERMANY

## Abstract

This study examines farmers’ intentions towards pro-environmental behavior in a famous tourist village in China called Guanshan, whose ecological environment is polluted. By adopting the empirically validated norm activation model (NAM) of Schwartz and merging it with Vroom’s expectancy theory, the current research aims to develop a refined framework for understanding the formation of and predicting changes in pro-environmental intention. Field surveys were conducted in Guanshan, which resulted in sample data consisting of 275 valid responses collected by the research team. We develop a refined model, including six latent variables and 24 observational items. The structural equation modeling results show that the final model enjoys a better predictive accuracy of pro-environmental intention than does the original NAM. The study also discovers that the motivational force of expectancy theory significantly influences pro-environmental intention, whose motivational force comes from the impact of valence and expectancy. The pro-environmental intentions of farmers are mainly affected by the environmental personal norm and by a certain degree of personal expectancy. The improvement of farmers’ pro-environmental intention needs be promoted in two approaches: the cultivation of personal environmental protection norms and the guidance of producing a desired expectation for pro-environmental intention.

## Introduction

China has many beautiful tourist villages; however, some of them are close to urban agglomeration and are thus affected by polluting industries, which significantly affects their natural ecological environment and reduces their local tourism value [1]. As some local governments in China are gradually becoming aware of the negative impact of environmental pollution on the sustainable development of rural tourism, they have formulated some effective governance policies to improve the ecological environment of polluted tourist villages; however, many of these policies have not achieved satisfactory results [2], and some farmers are not enthusiastic about the environmental protection provided [3]. In fact, local farmers in these villages are the most direct implementation force available to protect the environment, and their pro-environmental behavioral intention is the most important guarantee for improving these polluted tourist villages [4].

Guanshan village is located in the Changsha-Zhuzhou-Xiangtan city group and has many high-quality tourism resources. In the past, there have been many industries located in this area; some enterprises located around the rural tourist destinations in this urban agglomeration did not pay attention to the correct disposal of their industrial waste because of their lack of awareness of environmental protection, which caused heavy metal waste to leach into the soil of tourist destinations, thereby seriously exceeding the standard heavy metal content. This problem simultaneously affects the development of local agricultural production and tourism development, so it is urgent to pursue the environmental protection of polluted tourist destinations. However, the relationship between environmental protection and tourism development has often been debated by academics, tourism managers, and policy makers. Environmental protection mainly refers to taking administrative, legal, economic, scientific and technological measures to reduce the damage to the ecological environment, reduce the load of environmental utilization, and promote the self-organization of the ecosystem for the survival of natural creatures to maintain ecological balance [5]. Tourism development is generally planned and formulated by tourism experts, and these experts play a guiding role in protecting the ecological environment and improving farmers’ enthusiasm for environmental protection. There is a key issue in this debate that cannot be ignored; i.e., local farmers regard tourism as an important source of their livelihood income and thus, the intention of pro-environmental protection needs to consider the farmers’ livelihood interest expectations and the effectiveness of norm activation.

The research typicality of Guanshan Village lies in: it has a very developed rural tourism industry, which is developed by relying on local high-quality tourism resources and good ecological environment. A large number of farmers in Guanshan Village participate in the development of local rural tourism. However, some villagers destroy the local ecological environment, which is seriously harmful to the rural tourism. So, analyzing the environmental protection intention of local villagers in Guanshan Village is of great significance to the sustainable development of local rural tourism, and improving the villagers’ environmental protection intention is of great reference significance to the tourism development of relevant villages. To realize the sustainable development of rural tourism, it is necessary to seriously study the real pro-environmental intention of farmers to improve their enthusiasm for environmental protection. Mbaiwa and Stronza found that if farmers derive benefits from rural tourism resources, they will be obliged to use local tourism resources sustainably and show more positive pro-environmental intention [6]. Therefore, the purpose of this study is to examine a scientific theoretical framework based on various existing theories and contexts referred to as pro-environmental intention to explain how these pro-environmental intentions change and interact with each other.

In the previous tourism research, many achievements are absorbed by the eco-friendly tourism industry model and tourism service offerings, such as rural tourism, ecological tourism, low-carbon tourism, and green tourism [7]. Among them, rural tourism, as an eco-friendly industry, has been widely studied all over the world. However, these studies should pay more attention to focusing on the aggregate effect of different public behaviors. As a public power, farmers have an important influence on the development of tourism in rural areas. The acceptable consensus for sustainable rural tourism is that farmers’ supportive attitudes towards tourism development and their intentions to protect the rural environment should be taken into account simultaneously. Seetanah aggregated the dynamic impact of public actions on the economy and specifically considered that beneficial tourism behavior for island economies might have comparatively higher growth effects [8]. In fact, pre-environmental behaviors are not necessarily related to specific tourism services or products, but the pro-environmental behaviors of farmers can make a significant contribution to the environmental improvement of tourist destinations [9].

Peng pointed out that the evolution and characteristics of pro-environmental behavior at tourist destinations within this urban agglomeration tend to depend on local farmers [10]. Because the local environmental quality is closely related to livelihood development, these local farmers might show a positive attitude towards environmentally friendly behaviors. Another advantage of regulating the sample populations is that this study pays close attention to changes in the theoretical framework, while samples made up of very consistent members can help to reduce some potential model errors influenced by other mediators. Therefore, this study aimed to examine the behaviors of farmers located in tourist destinations. As the process of environmental protection around rural tourist attractions is influenced by various policies and administrative powers in China, the changing rules of different factors referring to environmental protection intentions based on activations and expectations have not yet been clarified. Hence, this embarrassing situation is not conducive to guide environmental protection or sustainable development of tourism. To improve the knowledge gap between attitude and intention in environmental protection theory, this study attempts to merge Schwartz’s norm activation model and Vroom’s expectancy theory to investigate the behavioral pro-environmental intention of farmers in tourist destinations whose ecological environment is polluted in China.

## Literature review

### Pro-environmental behaviors of farmers

The term "pro-environmental behavior" includes a wide range of behaviors, which can be interchanged with other words, such as ecologically friendly behavior, responsible environmental behavior, environmentally friendly behavior and green behavior. This study adopts Schwartz’s definition of pro-environmental behavior, which it discusses any behavior that protects the environment in the course of daily work, i.e., either a natural environment or an outdoor environment, to minimize the negative impact of human activities on the environment [11].

In the early study of pro-environmental behavior, social theoretical models based on the awareness of, attitudes towards and morality of environmental protection were widely used by researchers. This led to most environmental protection researchers attempting to increase the public awareness of rural environmental issues. Unfortunately, scholars later found that this approach is ineffective. The study of ecologically friendly behavior is mainly carried out in two aspects: altruism and self-interest. Tourism researchers who think that responsible environmental behavior has prosocial motivation usually adopt a theory based on ethics, such as Schwartz’s norm activation model (NAM) [12] or Stern’s recently introduced value-belief-norm theory (VBN) [13]. On the other hand, some researchers think that pro-environmental behaviors are driven by the interests of individuals. Thus, it is based on their high expectations of future benefits that farmers are motivated to protect the environment in specific areas [14]. The theory of self-interest is based on the premise that behavior is driven by desirable outcomes or expected rewards. Zhang Yuling (2014) further noted that the best approach is to mix the two aspects [15]. Keshavarz & Karami found an “attitude-behavior” gap among people of different professional identities. Nonfarmers, especially senior intellectuals, have a higher awareness of environmental protection. However, nonfarmers are less inclined to take positive actions than farmers [16]. Generally, farmers, and more precisely those who grow up in rural areas and rely on the rural ecological environment, will show more positive actions towards environmental protection.

As mentioned earlier, farmers empirically show a higher positive environmental attitude towards agricultural production practices. The awareness levels and norms of famers are found to be important predictors of pro-environmental intention because they serve as important emotional bonds between humans and ecological environment, and different degrees of participations may vary with various environmental protection intentions. Yu et al. found that if people feel more loving emotions towards a place, they are inclined to show a more positive responsible environmental behavior towards that place [17]. Lin et al. found that practitioners who feel a positive emotion of belonging to tourism are more inclined to show environmentally friendly behaviors at tourist destinations [7]. Agliardi & Agliardi pointed out that some respondents link environmental protection behavior with some constraints of time costs and higher spending; however, most of them show better environmental awareness and more positive environmental attitudes [18]. Olya and Akhshik also considered the barriers of pro-environmental behavior, including money cost, emotional input and sense of responsibility [9]. Given the background of this study, money cost, norms, and responsibility awareness may explain the environmental protection intention of farmers.

### Norm activation model

Schwartz originally developed the norm activation model, which involves altruistic behavior. "Personal norms" are the core element of this model. Schwartz states that "personal norms" are described "as feelings of moral obligation, not as intentions’’ [12]. The framework of the model aims to put forward a scientific understanding of individual supportive attitudes in the context of environmentalism. The support of the public is now regarded as one of the key powers to solving these social issues. The problem of public environmental protection is a large-scale problem that needs to be solved by social movements. Public environmentalism is progress that government leaders, tourism organization managers and farmers must realize the benefits of diminishing the adverse impact of human beings on the environment. These personal norms are used in the NAM to predict individual behavior, which is influenced by two factors: the feeling of responsibility for a specific behavior and the awareness that performing a particular behavior has a specific consequence [12]. Some studies have interpreted the norm activation model as a mediator model, while other studies have interpreted it as a moderation model. Researchers who consider the NAM as a mediator model have suggested that the awareness of consequences influences norms through ascribed responsibility. Researchers who consider the NAM as a moderation model have suggested that the influence of norms on behavior is moderated by the awareness of consequences and ascribed responsibility. This study deems the NAM as a mediator model; thus, we interpret that farmers should be aware of the consequences of environmental protection behavior before they feel responsible for it. Then, feelings of environmental responsibility will activate personal norms, and these norms will induce one’s intention of pro-environmental behavior [19]. Although effective pro-environmental policies need most of the related groups to be involved, Michel and Moser considered that the most critical group consists of grassroots farmers [20]. Hopper agreed with the assertion that to realize some movement goals, changes in individual-level behaviors among ordinary people are indispensable [21]. Most Chinese farmers have land to cultivate, such that the environmental protection behavior of farmers on their land can be regarded as behaviors related to the private field [22]. Such pro-environmental behavior in private-sphere environmentalism includes the adoption, opposition, and negation of the private sphere; organizational behavior; and products, services, and policies related to their lands that may have a negative impact on the environment. According to the NAM theory, pro-environmental behavior can be divided into four components: awareness of consequences, ascription of responsibility, personal norms, and intention of behavior [12]. Based on the limited scope of the current study, only activities related to collectively owned fields or daily agricultural work are analyzed and discussed. Schwartz considered that these four basic components are relevant to an ideal cross-situational goal with different importance as that of the environmental protection intentions required in the daily work of individuals or other social entities [12].

Awareness of environmental consequences (AEC) is the first construct in the NAM framework; it refers to the awareness that the problem related to environmental protection can be enhanced or diminished by the biosphere, people, and other species [23]. Environmental awareness is often influenced or promoted by different types of collective views and conclusions [12]. Awareness of environmental consequences has been widely considered by various disciplines, and its relationship as a predictor of environmental attitude is often supported by empirical evidence. Schwartz and Boehnke explained that the awareness of consequence construct is complex and can be composed of many variables [24].

Ascription of environmental responsibility (AER) is the responsibility that an individual’s behavior can either reduce or exacerbate the potential negative influence of consequences [25]. Montada and Kals found that the ascription of responsibility is the greatest contributor to farmers’ willingness to support an environmental protective policy [26]. Although the relationships between all the variables of the NAM often produce good statistical results, compared with other theories, that of predictive ability still has problems.

As the next construct, environmental personal norms (EPNs) are considered a social rule that guides how individuals should comply and are restricted by the ascription of responsibility [11]. Kaiser and Bogner found that normative behavior theory has a higher predictive power than many other theories regarding the environmental protection behavior of specific interviewers [27]. Gnoth subdivided tourism motivation according to personal norms. Their findings highlighted the potential for the segmentation of culture and that personal norms are sensitive to the use of cultural interpretation [28].

At the end of this cause-effect chain, the last construct examines pro-environmental intentions (PEI). Although we only measure individual’s intentions, some researchers have considered intention it is an important indicator of behavior. These individual intentions are assumed to be motivated by a personal norm, which is identified as some unavoidable obligation to carry out environmentally friendly behaviors, and the name of the component is used interchangeably [29]. On the whole, awareness of consequences, ascription of responsibility and personal norms are the three influence factor constructs of the norm activation model proposed by Schwartz; they are designed to effectively predict the personal intention of behavior [12]. This framework has been previously empirically verified by many researchers [25, 30].

The causal chain of the NAM can be viewed as follows: AEC→AER→EPN→PEI. In view of this analysis and evidence, the following hypotheses are proposed (H1-H3):

H1. Awareness of environmental consequences has a positive effect on the ascription of environmental responsibility.
H2. Ascription of environmental responsibility has a positive effect on environmental personal norms.
H3. Environmental personal norms have a positive effect on farmers’ pro-environmental intentions.

### Expectancy theory

Dunlap et al. explained that human beings need to protect the environment because they have the ability to disrupt the balance of unique nature; in addition, human society has growth constraints, such that humans have the right to adapt and rule the recognized parts of the natural world [31]. Bear argued that compared to environmentalists, the expectation of environmental protection reflects an individual’s view of the relationship between humans and the biosphere [32]. The intention of environmental protection can be predicted according to the types of values, which are an expression of individual expectations for environmental governance [25]. In the context of rural tourist destinations in which the ecological environment is polluted, Parijat and Bagga used expectancy theory to explore the formation of behavior choices about green tourism development [33]. Therefore, it is necessary to provide farmers with good expectations and active guidance to promote positive environmental protection intentions [34].

The classical theory of expectation motivation was proposed by Vroom [14]. His expectancy theory is rooted in the field of industrial and organizational psychology research. In the tourism industry, many researchers use this framework to capture tourism product suppliers’ motivations for destination image improvement, service attitudes, and community harmony. During this study, through a literature search, the author found that there was no previous clear application of expectancy theory in the field of analyzing behavioral pro-environmental intentions combined with NAM theory. However, after reviewing the NAM theory and other relevant theories, it was found that the two might be synergistic.

Expectancy theory is applied to explain the process by which individuals make decisions about various behavioral choices. The framework includes two different constructs: valence and expectancy. These two constructs determine the motivations that guide specific behaviors. The first component is valence, which determines to some extent the driving force or attractiveness of a specific outcome. The second component of expectancy theory is expectancy, which is the perception that a certain amount of performance will lead to the desired result. In other words, the more effort you put in, the more likely you are to get results. Finally, people believe that actions lead to positive expectations. In the study of Keshavarz & Karami, this theory was applied to predict how hard farmers support their environmental protective beliefs. The strength of the farmers’ work was affected by praises or rewards, whereas effectiveness with regards to how strong the effort should be ultimately ensured the desired results [16]. Therefore, the framework is expressed as follows:

Motivationalforceoffarmer’sintention=ThecombinedpositiveeffectsofValenceandExpectancy.

In the influence relationship, the two components are examined separately and then determine a motivational force together. However, the study suggests that these two components should be treated within a causal chain, leading to a motivated intention that is conducive to pro-environmental behavior when supporting the environmental protection behavior in the tourist destination. Since expectancy theory was put forward, there have been many explanations. The lack of clarity in explaining the interactions between these constructs of motivational force has prompted researchers to analyze how each construct affects other constructs.

However, testing the construct between causal relationships is often pursued. The above causal chain is supposed resembled the following: Valence→Expectancy→Motivational force.

In this case, valence is at the beginning of this chain, which is indicated by a farmer’s value for a particular outcome. The goal includes the desire for outcomes such as sustainable tourism development that can be achieved by improving the ecological environment. This leads to farmers having the expectation that the more effort they put in to expecting to reduce pollution, the greater their impact on improving the environmental quality will be. Individual contributions may be small, but they are effective overall, thereby emphasizing the role of effort; thus, it is assumed that valence has a positive influence on one’s expectancy. Therefore, it is an opinion that action will lead to the desired result. Expectancy can affect environmental intention. From the discussion of expectancy theory, two assumptions are put forward:

H4. Valence has a positive effect on expectancy.
H5. Expectancy has a positive effect on farmers’ intention to behave pro-environmentally in tourist destinations in which the ecological environment is polluted.

### Convergence of the two theoretical frameworks

Through a large number of studies that have focused on pro-environmental behavior research, we can find that discussions of the individual sociopsychological construct are more predictive towards pro-environmental behavior than are discussions focusing on the sociopopulation demographic background [35]. Variables such as awareness, responsibility, norms, and behavioral intention of environmental protection empirically refer to personal feelings and ways of thinking and ultimately affect solutions to environmental problems [36]. The theory has been confirmed, and its effectiveness has been tested [19]. In addition, compared to other theories, its pro-social construct of the NAM model has led researchers to agree that it has superior predictive power. However, the NAM has been studied along with other well-known attitude theories, such as Stern’s value-belief-norm theory [37]. Van and Meertens suggested that the combination of pro-social and rational choice theories can produce higher predictive ability [38]. Park and Ha combined the NAM and planned behavior theory into a single theoretical framework [39]. Their findings support the suggestion of Van and Meertens; the model shows a better precision regarding consumer recycling behavior than the independent NAM framework. The successful application of the merged model in interpreting individual environmental behavior demonstrates the validity of the NAM. In contrast, adopting the expectancy theoretical framework helps improve the performance of separate NAMs. However, while expectancy theory is a rational research framework that has been widely accepted worldwide, it still receives some criticism from researchers [34]. A prominent criticism of this theory is its lack of consideration for social impact [40]. By combining expectancy theory with the NAM model, it is assumed that this limitation will be reduced. The NAM originated from a theory that studies social movements. These measurement items involve individual awareness, responsibility, and personal norms about the behavioral intentions for environmental protection, which affect both human society and the local environment. The important view is that expectations for a better living environment and tourism livelihood income growth may affect one’s eagerness to protect the local environment [41]. Yuan et al. considered that personal norms are usually formed by social interaction, but one’s final decision is not simple [42]. Sometimes, one’s desired expectations may affect the amount of the individual’s actual effort and ultimately affect the environmental norms. Although expectancy theory measures a narrower range of variables than the NAM, the NAM does not need to consider the frequency of actions. Improving farmers’ intentions to support the protection of the ecological environment of tourist destinations requires repeated efforts rather than one-off actions. The impact of norms is essential to promote broad improvements in pro-environmental behaviors. Based on these discussions, the link between the two theories is hypothesized as follows:

H6. Valence positively affects awareness of environmental consequences.
H7. Expectancy positively affects environmental personal norms.

**Fig 1:**
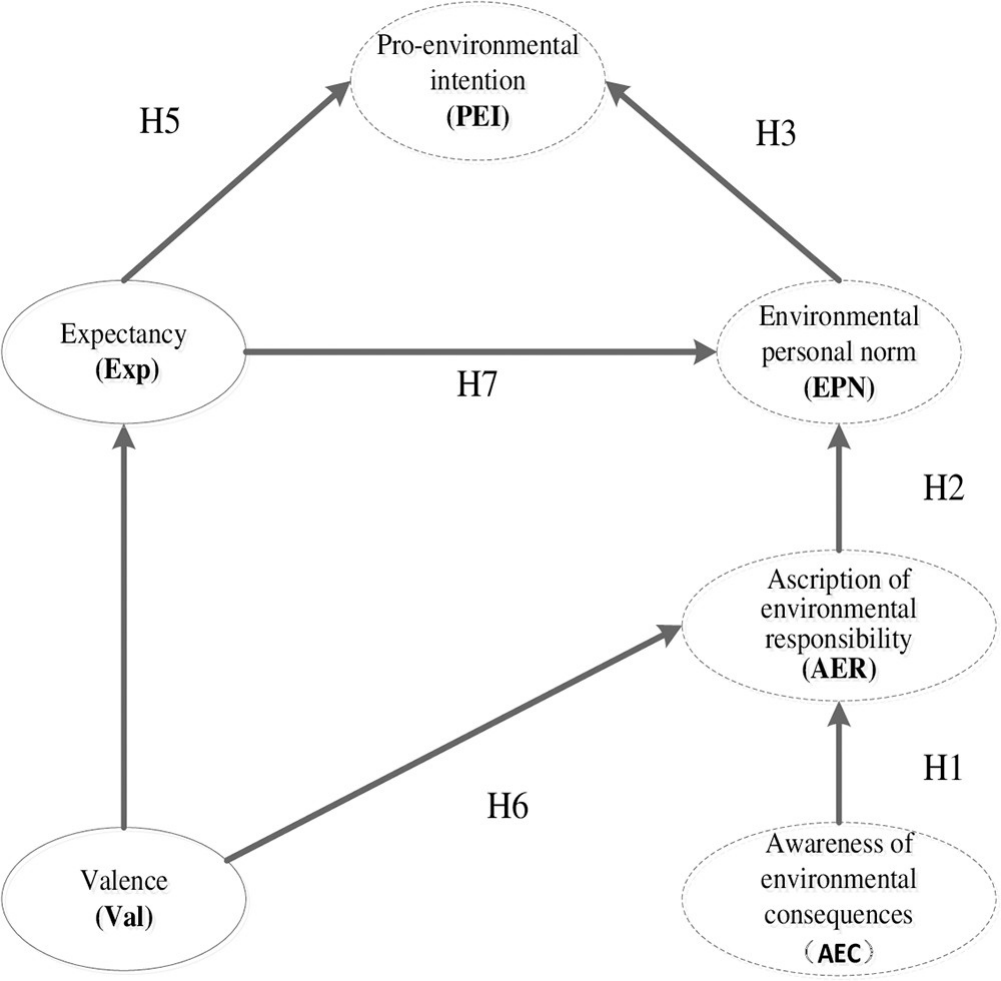
The proposed research model. Constructs with dashed boxes represent the norm activation model (NAM).

The proposed theoretical model includes a total of 6 constructs and seven hypotheses. The interactive relationships between the six constructs are illustrated in Fig 1.

## Methodology

### Study context and case selection

Guanshan village is located in the northeast portion of Ningxiang district, Changsha city, Hunan Province, China. It is a world-renowned Chinese rural tourist destination that is 24 kilometers away from Changsha city in the east. It is named after Guan Yu, who was the general of *the Shu dynasty* during the Three Kingdoms Period (the Three Kingdoms were composed of the states of *Wei*, *Shu and Wu*) and was stationed troops there. Guanshan, as a world-famous rural tourist destination with high-quality ecological environments that are being gradually destroyed, has many types of high-quality tourism resources.

The Guanshan Village is close to the metropolis of Changsha in the centre of China. As a large-scale rural tourism scenic spot, it won the honorary title of "The most charming leisure village in China" in 2012, which is the first famous rural tourism in Hunan Province to win this honorary title. In addition, as a whole scenic area, Guanshan Village has been officially approved by the National Scenic Area Evaluation Committee on September 18, 2013, and successfully established as a national AAAA scenic spot (the highest scenic spot in China is 5A, and Guanshan Village is one of the most famous rural tourism villages in Hunan province at that time).

Due to the rapid development of rural tourism, a large number of farmers in Guanshan Village have joined the rural tourism development industry. However, due to the overloading of local rural tourism services and the limited rural ecological carrying capacity, the contradiction between rural tourism supply and demand is prominent. Moreover, some local villagers even directly destroy the local ecological environment in order to obtain the income from rural tourism. It can be seen that the phenomenon of rural tourism in Guanshan Village is relatively developed, but the local ecological environment is also at risk of being destroyed. It can be seen that the analysis of the intention of local farmers in tourist destinations who participate in tourism for the protection of rural ecological environment is very critical.

**Fig 2:**
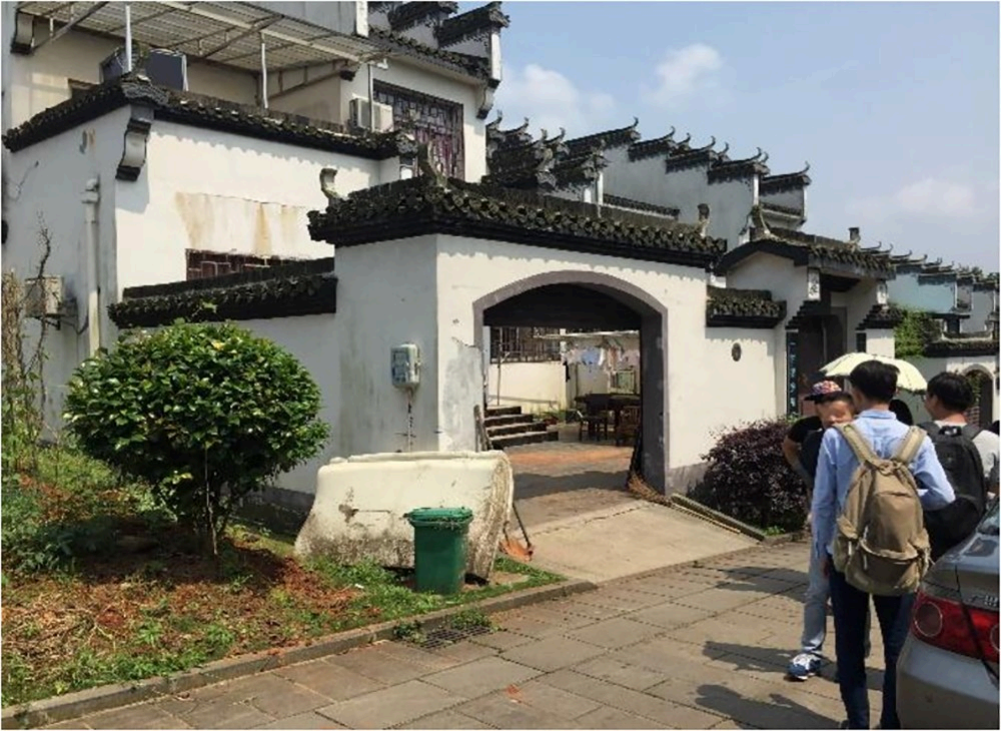
Landscape of Guanshan village. Source: Photographed during field research.

**Fig 3:**
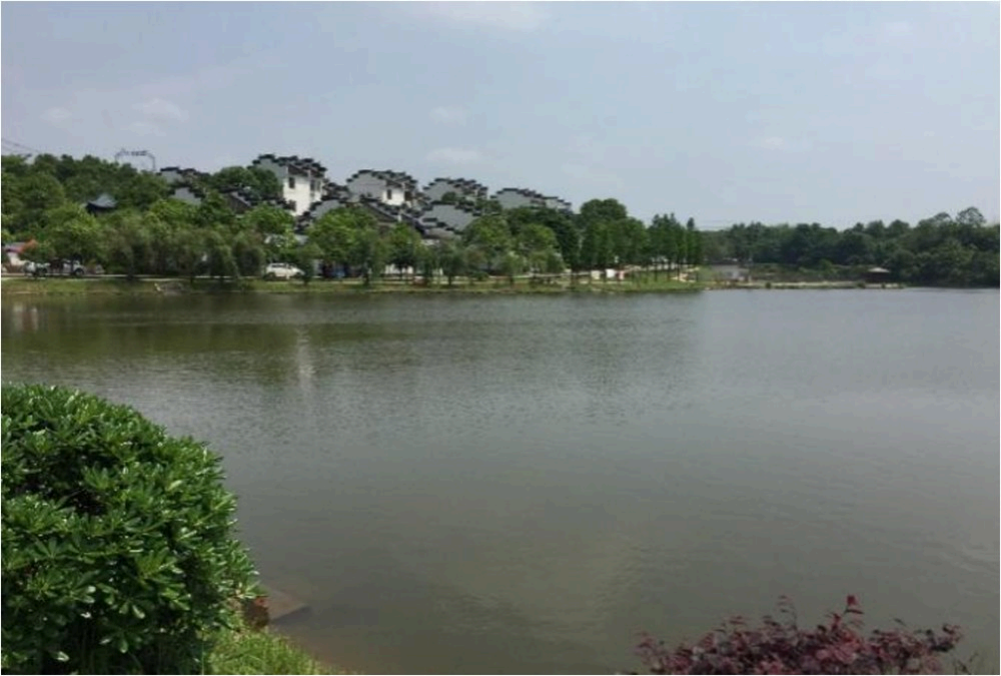
Appearance of Guanshan village. Source: Photographed during field research.

There are two characteristics of the rural tourism found in Guanshan; one is the farmhouse tourism area, which was built in 2004. The farmhouse tourism model adopts a variety of mixed modes, such as "theme picking + agricultural experience + science education + green catering", and it takes the Fangyuan Mountain villa as the core area. Relying on five modern leisure agricultural bases, Guanshan can provide local agricultural products to foreign tourists. Another characteristic of rural tourism in Guanshan is its high-quality natural landscape and local historical buildings (see Fig 2). However, due to the increase in the number of foreign tourists and the excessive service pressure of these tourists, a large amount of sewage and domestic garbage is generated and directly flows into the local water body, causing damage to the local water body landscape (see Fig 3). At the same time, local farmers’ domestic wastewater is discharged arbitrarily, there is an excessive application of chemical fertilizers, garbage is incinerated in the core area of the tourist destination, and a massive amount of land is occupied for tourism reception. These behaviors have seriously damaged the quality of the natural ecological environment of the local tourist destinations, resulting in a decline in the tourist attractions in the recent period and a recent reduction in the number of tourists. Moreover, the tourist satisfaction score of Guanshan found on the internet has also decreased significantly. To improve the local rural tourism, it is important to study how to stimulate local farmers’ pro-environmental intentions.

### Data collection

Data were collected by the author and five undergraduate students majoring in human geography and urban planning from Hunan Agricultural University in 2020. The six investigators conducted field surveys in Guanshan village from May 1 to May 3, 2020. The author provided training and guidance to the investigators before their departure. Before issuing the questionnaire, respondents were asked if they were farmers in Guanshan village, whether they were affected by the harm of environmental pollution, whether they were currently engaged in tourism, and whether they agreed to fill the questionnaire anonymously for an academic study. Questionnaires were issued only after verbal agreement by the farmers. As the overall identity of the sample is relatively consistent, thereby providing a fairly homogeneous sampling profile for research and analysis, the collected data have high internal validity. A total of 305 responses were gathered for the survey. To maintain the credibility of the responses, if more than 10% of the data in a questionnaire’s responses were missing, the questionnaire was removed. Therefore, 9 surveys were deleted at the beginning of the process. The standard deviation of each response was then calculated to remove the unengaged responses if a case with a value less than 0.5 was noticed when reviewing the data for patterns exhibiting the nonengagement of the respondents. Thus, 21 responses are deleted due to unengaged performance. As a result, the actual number of valid responses was 275, and the effective rate of questionnaires was 90.16%. For a few missing data points, this study used the series mean to replace and then further analyze these valid responses.

### Measurement instruments

The scale and items used in the measurement study were obtained from previous studies validated in various research contexts. Vroom’s expectancy theory is widely used in the study of the behavioral will of community farmers around tourist destinations. The use of a validated scale provides a solid theoretical basis; it also allows for accurate comparisons with other research results. Thus, the related items were modified to make them fit the setting of the current study. The measurement items of pro-environmental intentions, simulated by the final construct, are adopted from previously widely accepted research achievements, including *I would give priority to accepting the guidance of agricultural experts on how to reasonably carry out agricultural activities around the tourist destination*; *I would sacrifice my rest time to promote the environmental protection policy; I would persuade others to protect the ecological environment of tourist destinations; I would spend more money to improve the tourism service facilities in order to reduce environmental pollution*, *I would learn relevant environmental protective skills and knowledge*, *etc*. Additionally, the tone and language of the questionnaire used in the field survey was discussed to adapt an understanding of the habits of the target sampling population, i.e., farmers with low education levels. The purpose of this approach was to make it easier to understand the measurement items to minimize the number of invalid responses and reduce the potential errors. In this study, a five-point Likert scale was used for all measured variable items, and there was no coding problem. The scale of the questionnaire’s scores ranged from "extremely disagree" (1) to "extremely agree" (5). Before the final version was confirmed, the team invited some experienced academics, environmental experts, and undergraduates to conduct pretests and assessments. Based on their feedback, minor adjustments were made to the formatting, grammar, wording, experience, and visual construct of the respondents. Since the sample consisted of farmers engaged in tourism in Guanshan village, the final version of the questionnaire was presented in Chinese to help the farmers, many of whom do not have enough education to understand or read English. To avoid bias and personal input, the translators knew nothing about the research beforehand. In addition, to ensure successful expression, the results were in good agreement with the Chinese language scale version. A cover paragraph was placed at the beginning of the questionnaire, which briefly summarized the purpose of the study and briefly described the discipline of this study. Finally, the questionnaire included demographic questions; however, sensitive personal issues and personal contact information were not involved.

### Tools for analysis

PASW Statistics 26 and AMOS version 21 were used for data analysis in this paper. In recent research, the combined use of SPSS and AMOS has been widely popular, mainly due to the combination of being easy-to-visualize and user-friendly when dealing with conceptual models. In the first stage, the adequacy of the proposed measurement model was analyzed, and the author used confirmatory factor analysis to assess the validity and reliability of the proposed research model. After achieving satisfactory fitting results, the second stage examined the proposed hypotheses in this study by providing path coefficients for the different hypothetical relationships. A model comparison between the existing model and the model proposed in this study was used to verify the constructed model, that is, to establish an adaptive and robust intention model to explain the environmental protective intentions of farmers in tourist destinations.

## Results

### Sample profile

Farmers of GuanShan village comprised the research sample, and they regard tourism as the main source of their livelihood income. A summary of the study sample is shown in Table 1.

**Table 1:** Demographic characteristics of valid responses.

Variable	Category	Distribution (person)	Valid percentage (%)
**Gender**	Male	162	58.91
	Female	113	41.09
**Age**	Mean	50.47	
	18–24	10	3.64
	25–35	16	5.82
	36–50	137	49.82
	51–60	72	26.18
	over 60	40	14.55
**Education**	primary school or below	92	33.45
	junior high school	106	38.55
	senior high school	53	19.27
	college degree	10	3.64
	bachelor’s degree	14	5.09
	graduate degree or above	0	0
**Job types**	full-time work	212	77.09
	part-time work	63	22.91
**Annual income** (after taxes) exchange rate in January 2020 ($1 = ¥6.9326)	Under $1,731	7	2.55
	$1,732-$3,462	67	24.36
	$3,463-$5,193	37	13.45
	$5,194-$8,655	86	31.27
	$8,656-$14,425	51	18.55
	$14,426-$28,849	15	5.45
	$28,850-$43,274	8	2.91
	Over $43,275	4	1.45

From Table 1, it can be seen that there are 275 valid samples. A total of 58.91% of the valid survey respondents were male (162), and 41.09% of the responses were female. The ratio of males to females was close to 3:2, which is in line with the local village officials’ judgment of the population. The average age of the 275 valid respondents was 50.47 years old. Thus, the respondents were mainly from the middle-aged and elderly populations; a total of 137 persons were aged between 36 and 50, accounting for 49.82% of the sample. Seventy-two persons aged between 51 and 60 made up 26.18% of the sample. The majority of the respondents had a mid- or low-level education; a total of 92 farmers had only a primary school education or below, accounting for 33.45% of the sample, while 106 farmers had a junior high school degree, comprising 38.55% of the sample, and 53 farmers had senior high school degree, accounting for 19.27% of the sample. Approximately 77.09% of the respondents reported that they were engaged in tourism full-time, while only 22.91% of the respondents reported that they were engaged in tourism part-time. From the perspective of annual income (after taxes) obtained from local tourism, most of the farmers’ reported incomes were not high compared to the per capita disposable income of Chinese residents (the per capita disposable income of Chinese residents was $4,433 in 2019). The specific differentiations were as follows: 24.36% of the farmers made between 1,732 dollars and 3,462 dollars (12001 to 24000 yuan); 13.45% of the farmers made between 3,463 dollars and 5,193 dollars (24,001 to 36,000 yuan); 31.27% of the farmers made between 5,194 dollars and 8,655 dollars (36,001 to 60,000 yuan); and 18.55% of the farmers made between 8,656 dollars and 14,425 dollars (60,100 to 100,000 yuan) (the average exchange rate of RMB against USD was 6.9326 yuan to 1 US dollar in January 2020).

It can be inferred that these responses about after-tax income distribution from those engaged in local tourism are relatively optimistic compared with the income level of other local farmers in Guanshan village. Under the promotion of environmental protection in rural areas, the relatively good income related to rural tourism will enhance farmers’ expectations for their future life, which will also prompt farmers to make positive pro-environmental intentions or behaviors. It can be inferred that the sustainable development of rural tourism closely refers to farmers’ intention of pro-environmental behavior. The ratio of the effective sample responses (275 valid samples) to the 24 observational items is much greater than 5:1, and the number of valid responses is more than 200, which can significantly reduce the risk of the nonnormal distribution of samples [43]. Therefore, the 275 valid responses cover the main attribute characteristic information of the sample, and the data distribution is in line with the actual situation.

### Confirmatory factor analysis (CFA)

Testing the intention model goodness of fit is the first stage of the two-step approach proposed by Cheung. Mike and Chan. Wai [44]. Before the CFA, the research data were screened to check for possible violations of the proposed seven hypotheses. The test showed no apparent violation of the assumptions. The value of skewness ranges between -1 and 1, and the measurement items did not exceed this value range. Therefore, the items were not considered to violate the assumptions. The kurtosis values of the measurement items were all below the recommended threshold of 3. Further examination revealed no violations related to homoscedasticity, linearity, or multivariate normality.

The CFA factor analysis method is a maximum likelihood method, and the test results show that the proposed model has a goodness of fit (χ^2^ = 566.559, df = 218, p<0.001, χ^2^/df = 2.599, IFI = 0.913, TLI = 0.888, NFI = 0.865, PGFI = 0.618, RMSEA = 0.076, CFI = 0.911). All the indices showed that the measurement model has good acceptability. According to Gholami, Asli et al., the CFI scores of the 275 valid samples were all above 0.90 for the 24 measurement items [45]. Each latent variable involves multiple measurement items; thus, to verify the internal consistency, the composite reliability test was carried out between different latent variables. The test scores showed that the values of each indicator were larger than most the minimum threshold of 0.70 accepted by most scholars, and their scores ranged between 0.899 and 0.912. In Table 2, the validity tests of the constructs are explained; the convergence validity of the different latent variables displays an average variance extraction (AVE) score between 0.511 and 0.688, which is larger than the generally accepted minimum requirement of 0.5 [45]. Finally, the AVE scores are larger than the correlation between the study constructs, providing evidence for discriminant validity [46].

**Table 2:** AVE, ASV, correlation, reliability, mean and standard deviation values.

	AEC	AER	EPN	PEI	Val	Exp	AVE	ASV
AEC	0.748 ^a^	0.239 ^b^	0.067	0.060	0.024	0.012	0.559	0.080
AER	0.489 ^c^	0.719	0.256	0.333	0.015	0.112	0.517	0.191
EPN	0.259	0.506	0.829	0.518	0.136	0.281	0.688	0.252
PEI	0.245	0.577	0.720	0.810	0.084	0.286	0.657	0.256
Val	0.155	0.122	0.369	0.290	0.715	0.146	0.511	0.081
Exp	0.108	0.335	0.530	0.535	0.382	0.779	0.607	0.167
Mean	2.403	3.608	4.178	3.344	3.650	3.640		
SD	0.935	0.995	0.810	1.093	0.928	0.917		

Note 1. Goodness-of-fit: χ^2^ = 566.559, df = 218, p <0.001, χ^2^/df = 2.526, RMSEA = 0.075, CFI = 0.911, IFI = 0.913.

Note 2. AEC = awareness of environmental consequences, AER = ascription of environmental responsibility, EPN = environmental personal norm, PEI = pro-environmental intention, Val = valence, Exp = expectancy.

^a^ Composite reliability, which is the square root of average variance extraction.

^b^ Squared correlations.

^c^ Correlations.

### Structural equation modeling

The second stage of the two-stage approach proposed by Cheung, Mike W-L and Chan, Wai is to examine the structural model in the study [44]. Structural equation modeling (SEM) analysis was used to test the goodness-of-fit of the proposed model, and the results indicate satisfactory statistics (χ^2^ = 583.536, df = 231, p<0.001, χ^2^/df = 2.526, IFI = 0.911, TLI = 0.893, NFI = 0.861, PGFI = 0.650, RMSEA = 0.075, CFI = 0.910). Based on the indices recommended by Hair (2014) [46], the fit of the hypothesized model was satisfactory. However, some suggestions concerning the modification indices from the AMOS output showed significant improvements in the proposed model. After adding one path from the ascription of environmental responsibility to expectancy, the goodness-of-fit statistics improved significantly. The originality of the proposed model of farmers was minimally influenced (χ^2^ = 528.887, df = 217, p<0.001, χ^2^/df = 2.437, IFI = 0.922, TLI = 0.900, NFI = 0.904, PGFI = 0.620, RMSEA = 0.072, CFI = 0.921). For this purpose, the structural model after the path adjustment was made was taken as the final model for in-depth analysis and discussion in the current study. The purpose of the study is to develop a theoretical framework to help researchers to explain the pro-environmental intentions of farmers in tourist destinations where the ecological environment is polluted. Therefore, the adjusted final model was compared with the NAM’s original framework (χ^2^ = 298.173, df = 102, p<0.001, χ^2^/df = 2.923, IFI = 0.936, TLI = 0.913, NFI = 0.905, PGFI = 0.701, RMSEA = 0.084, CFI = 0.935). The final model showed a higher goodness-of-fit than the proposed model mentioned above (χ^2^/df = 2.437) compared to (χ^2^/df = 2.526), and it also showed a better goodness-of-fit than the original NAM (χ^2^/df = 2.923). The results from a chi-square test of the final model and the proposed model revealed that there were significant differences between them (Δχ^2^ = 37.672, Δdf = 1, p < 0.01). The goodness-of-fit of the final model was also relatively better than that of the original NAM. Moreover, these two models were distinctly different (Δχ^2^ = 230.714, Δdf = 115, p < 0.01). The final model has a relatively excellent ability to predict farmers’ environmental protective intention (R^2^ = 0.727) compared with the original NAM (R^2^ = 0.684) and the proposed model (R^2^ = 0.702). From the contrastive results, the final model has a 6.3% stronger predictive power of intention than the original NAM alone. A comparison summary of the structural models is displayed in Table 3.

**Table 3:** Results of the structural model comparisons.

Goodness-of-fit	NAM	Proposed model	Final model
χ^2^	298.173	566.559	528.887
*df*	102	218	217
χ^2^/df	2.923	2.599	2.437
RMSEA	0.084	0.076	0.072
CFI	0.935	0.911	0.921
IFI	0.936	0.913	0.922
TLI	0.913	0.888	0.900
NFI	0.905	0.865	0.904
PGFI	0.701	0.618	0.620
PEI’ *R*^*2*^ (Adjusted)	0.684	0.702	0.727

Note 1. Chi-square difference test between the final model and the hypothesized model:

Δχ^2^ = 37.672, Δ*df* = 1, p < 0.01.

Note 2. Chi-square difference test between the final model and the NAM theory:

Δχ^2^ = 371.316, Δ*df* = 230.714, p < 0.01.

The relationships among the different constructs were examined as hypothesized. Then, an additional significant path was discovered from the analysis of the structural equation modeling.

Table 4 shows a summary of these findings; awareness of environmental consequences was found to be significantly related to the ascription of environmental responsibility. Ascription of environmental responsibility was found to be significantly related to environmental personal norms and expectancy. Valence was found to be significantly positively correlated with expectancy. Expectancy was found to be positively correlated with environmental personal norms. Expectancy and environmental personal norms were found to be significantly positively correlated with pro-environmental intention. Therefore, hypotheses 1, 2 and 3 were supported. As expected, the relationships among the NAM constructs were significantly positively correlated. Hypotheses 4 and 5 were also supported, as expected. Hence, the two constructs involved in expectancy theory were significantly positively correlated. Finally, we need to focus on the two hypothesized relationships that link the NAM framework to expectancy theory (H6 and H7). While hypothesis H7 displayed positive relationships and was thus supported, hypothesis H6 was found to have an insignificant positive correlation. Therefore, hypothesis H6 was rejected.

**Table 4:** Standardized parameter estimates of the different influence paths.

	Standardized estimate	*t*-value	Hypothesis
H1: AEC→AER	0.502	6.899*	Supported
H2: AER→EPN	0.328	4.536*	Supported
H3: EPN→PEI	0.739	8.964*	Supported
H4: Val→Exp	0.397	5.955*	Supported
H5: Exp→PEI	0.173	2.977*	Supported
H6: Val→AER	0.016	0.336	Rejected
H7: Exp→EPN	0.473	6.463*	Supported
D1: AER→Exp	0.325	4.548*	Discovered
Total impact on pro-environmental intention:	AEC = 0.207, AER = 0.412, EPN = 0.739, Val = 0.214, Exp = 0.523		
Goodness-of-Fit Statistics for the final model:	χ^2^ = 528.887, df = 217, p<0.001, χ^2^/df = 2.437, IFI = 0.922, TLI = 0.900, NFI = 0.904, PGFI = 0.620, RMSEA = 0.072, CFI = 0.921.		
Total variance explained:	R^2^ of PEI = 0.727, R^2^ of AER = 0.255, R^2^ of EPN = 0.443, R^2^ of Exp = 0.286.		

Note.

*p < 0.01

**Fig 4:**
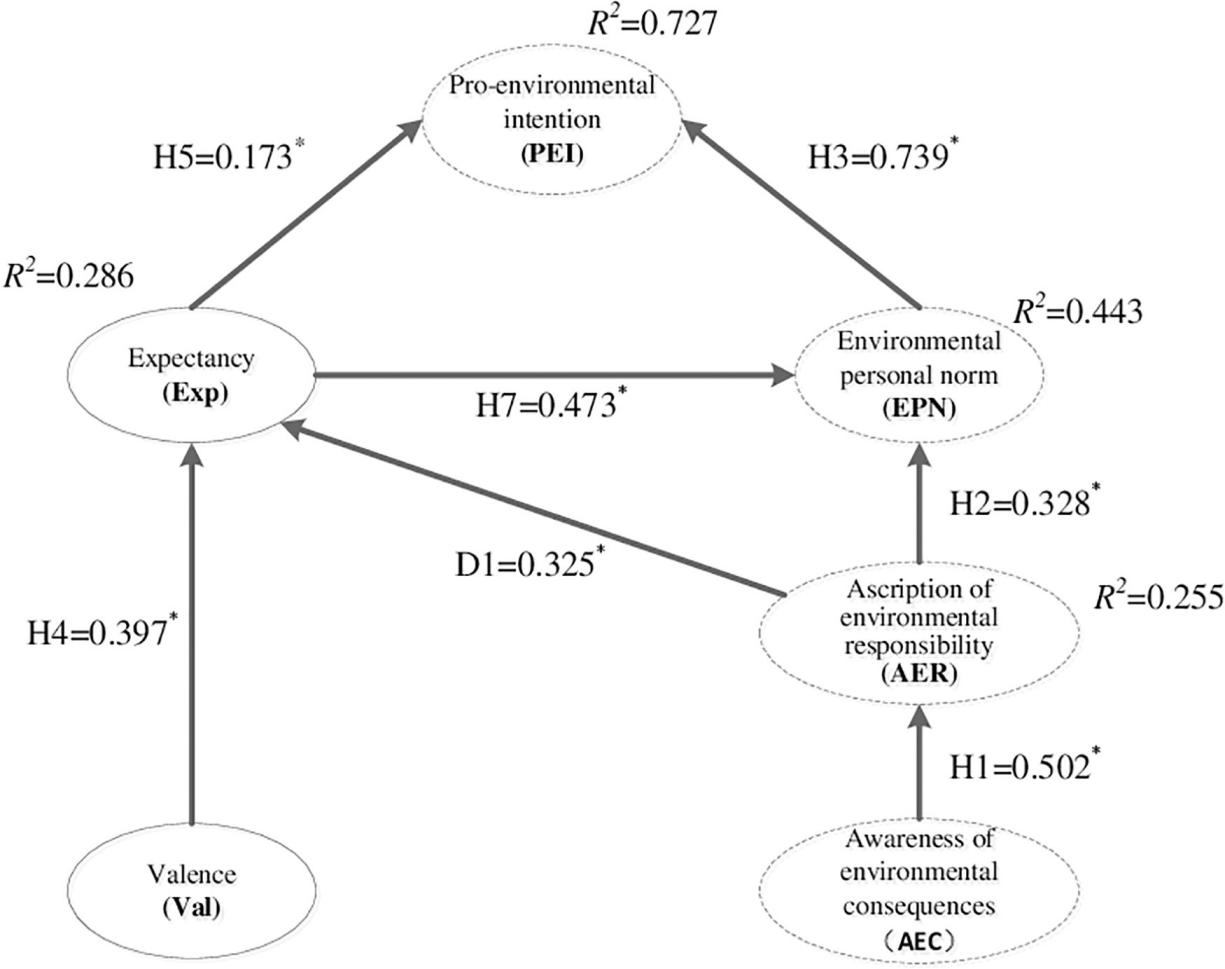
Results of the final model of the structural equation modeling. *p<0.01, constructs with dashed boxes represent the NAM theory.

As mentioned above, the modification index analysis of the different influence paths referred to structural equation modeling and indicated the discovery of an additional relationship not found in the initial hypotheses. The study attempted to introduce this additional relationship into the final model, and it will be retained for further discussion with other models in future studies. The additional discovered relationship (D1) based on the proposed model was between the ascription of environmental responsibility and expectancy. Due to the inclusion of the newly discovered path, compared with the proposed model and the norm activation model, the final model was demonstrated to be improved in regard to model fit statistics. Hence, this study has opted to retain the discovered path. Fig 4 shows the results of the final model and the structural equation modeling analysis.

From the total variance explained by the final model, the results indicated that 25.5% of the ascription of environmental responsibility was explained by awareness of environmental consequences. Valence then explained 28.6% of the expectancy. As we expected, the incremental increases referred to the R^2^ values in the ascription of environmental personal norms (44.3%), indicating that the total variance was explained by the antecedent variables. Finally, 72.7% of pro-environmental intention was explained by environmental personal norms and expectancy.

The indirect impacts among those constructs were assessed, and their results were as follows. Except for EPN, all the other factors had a positive and significant indirect impact on farmers’ intentions of pro-environmental behavior, namely, awareness of environmental consequences (β = 0.207, p<0.01), ascription of environmental responsibility (β = 0.412, p<0.01), valence (β = 0.214, p<0.01), and expectancy (β = 0.350, p<0.01). Environmental personal norms did not show a significant indirect impact on farmers’ intention of pro-environmental behavior. Additional evidence of mediation effects also shows that expectancy was indirectly affected by valence (β = 0.005, p<0.05) and awareness of environmental consequences (β = 0.163, p<0.01). Environmental personal norms were indirectly affected by valence (β = 0.195, p<0.01), awareness of environmental consequences (β = 0.242, p<0.01), and ascription of environmental responsibility (β = 0.154, p<0.01). Pro-environmental intention was also indirectly affected by valence (β = 0.214, p<0.01), expectancy (β = 0.350, p < 0.01), awareness of environmental consequences (β = 0.207, p<0.01), and ascription of environmental responsibility (β = 0.412, p<0.01). Details of the indirect impact assessment are shown in Table 5.

**Table 5:** The assessment of indirect impact.

Indirect effect of	On				
	AEC	AER	Val	Exp	EPN
Ascription of environmental responsibility	—	—	—	—	—
Environmental Personal Norm	0.242**	0.154**	0.195**	—	—
Expectancy	0.163**	—	0.005*	—	—
Pro-environmental intention	0.207**	0.412**	0.214**	0.350**	—

Note.

**p < 0.01,

*p < 0.05.

## Discussion

This study attempts to fuse two classical theories in the field of behavioral intention research in regard to tourist destinations where the ecological environment is polluted. In past academic achievements, the NAM has a relatively significant predictive ability in the aspect of pro-environmental behavior [47]. In some other areas, expectancy theory is also used to predict pro-environmental behavior in a specific context with relevant stakeholders [48]. This study merged the NAM framework with expectancy theory to develop a more accurate and robust model to scientifically predict farmers’ pro-environmental intentions in the tourist destination.

Although the original NAM studies intentions after the process of awareness of consequences, the attribution of responsibility, and personal norms, as well as analyzes daily pro-environmental behaviors and their aggregate impacts, it cannot effectively measure an individual’s "effort." However, while expectancy theory has been criticized by some scholars since it was developed as a theory, its versatility has been affirmed [33]. Expectancy theory focuses on an individual’s "effort" in the process of application [49]; for example, "The correct ascription of environmental responsibility helps farmers to expect the desired outcomes", and "The higher your expectations are, the more positive behavioral intentions to achieve the tasks of the environmental protection you have." Hence, the study results of these hypotheses prove the rationality of the merger of these two theories.

In the various constructs of expectancy theory, the causal relationships expressed by H4 and H5 are as significant as the proposed hypothesis. The findings show that valence (the value the farmer personally places on rewards of pro-environmental behavior) is an important premise of efforts. That is, "effort" regarding the pro-environmental intention may be influenced by the desired outcomes. However, from the perspective of the NAM model, the activation of pro-environmental intentions is usually predicted by personal norms and ignores the guidance and expectation of intentions and behaviors [50].

Since two constructs were added to the model to measure intentions from the influence factor perspective of "effect" with respect to expectancy theory, the predictive power of measurement intention was significantly improved compared to that of the original NAM framework; this outcome supports the researchers’ emphasis on how effort might have a positive effect on the predictive power of the model. As expected, when farmers feel that their pro-environmental behavior may contribute to some improvements of the local environment, it has a positive effect on their intention to support environmental protection when developing rural tourism and adopt sustainable measures.

Based on the findings of Robert L. Shrigley et al., who researched the gap between an individual’s attitude and behavior [51], some respondents in the questionnaire survey felt that they should persuade others to care for the ecological environment, citing that "I am willing to persuade others to protect the ecological environment of tourist destination." We found that these results support a potential approach to improving individual environmental protective behaviors. This approach avoids positioning the pro-environmental behavior and their intentions as a daily requirement or a mundane convention, but it promotes the outcomes (the ecological environment should be improved) of a desirable pursuit.

The influence relationships among the NAM constructs showed significant correlations, consistent with Shah et al.’s [52] and San Martín Héctor et al.’s results [53]. As expected from the initial hypothesis, there was no significant link found between valence and the ascription of environmental responsibility. The reason for this deviation from the initial hypothesis could be due to other peculiar phenomena that cannot be recognized or explained by the study under the influence of special backgrounds and circumstances, which is not the result of the validity of the model itself. One of the possible reasons may be that farmers are not very concerned about their social dominance, individual rewards, awareness of negative consequences, or utilitarianism. Through the empirical findings, it was indicated that the awareness of the environmental consequences, ascription of environmental responsibility, and environmental personal norms make up an important basis to explain the pro-environmental behavior of individuals. Compared with some other studies, the conclusions are slightly different. Kiatkawsin found PPN to have the highest R^2^ value among the variables of the final model [40], whereas personal norms were the biggest influencing factor in this empirical study, which further illustrates how behavioral intentions are made within two different contexts. Suppose that personal norms drive environmental protection intentions and corresponding behaviors and that these behaviors often involve minimal intention-making. At the same time, the participative intention of behaving pro-environmentally does not happen frequently. Hence, it involves a complex and deeper intention-making process. It also shows how the respondents feel a positive effect on the environment in this study. The measurement scores of total impacts on behavioral intention (the final model) are further supported as evidence in the daily practice of environmental protection. Additionally, the results suggest that personal norms exert the greatest amount of total impact on various factors affecting environmental protection intention, which is consistent with the conclusions of Bamberg and Moser, who considered that personal norms were the most important predictor of individuals’ environmental protection intentions [50].

As we hypothesized, the relationships between the two models were proven to be positively significant. A positive H5 was parallel with previous research conclusions [49]. It can be safely explained that farmers who have a positive intention of environmental protection usually expect to have a good tourism livelihood income and a beautiful natural tourism environment. The discovery of one path relationship (D1) helped us to deepen the previous discussion of how personal norms play an important role in predicting farmers’ behavioral intentions. These analyses and findings illustrated not only that pro-environmental behavior starts from the awareness of environmental consequences and valence concerning the environment but also that such behavior can be influenced and then generated.

Finally, testing the final construction details revealed some interesting results; the emergence of one pattern in particular suggested that farmers’ implementation of corresponding environmental protection actions potentially involves increasing farmers’ cost of environmental remediation and may even significantly reduce their income from tourism. The farmers’ actual actions and real intentions to protect the environment are less satisfactory than our initial model estimations. This new finding is consistent with Liu C et al.’s argument that a lack of money or reducing the previous income levels is one of the obstacles to supporting environmental protection behaviors whether they are rational or not [54]. Given that the research sample consisted of farmers (who are in fact not very rich), tourism livelihood obstacles were reflected in the results. Therefore, it is a pragmatic prerequisite for environmental protection to address the source of environmental money and protect farmers’ tourist income from the obvious negative impacts on tourist destinations where the ecological environment is polluted.

The research results also provided basic approaches regarding how to promote pro-environmental behavioral intentions and the main constructs of the final model that need to be manipulated in this environmental protection [50]. In practice, the farmer should first look at the awareness of environmental consequences and the valence of protecting the environment. Heesup et al. suggested that the awareness and valence of individuals’ environmental protection are important factors in tourist destinations [55]. Therefore, some local governments have improved the rewards on environmental protection behavior and the corresponding environmental protection education. They have realized that the awareness of environmental responsibility could stimulate individuals’ protective norms [56]. The study further found that the effective implementation of protective schemes must pay attention to the influence of environmental norms.

The use of environmental norms including comparing an individual’s behavior with that of other farmers’ (i.e., “I consider that some farmers in the tourist destination need to learn relevant environmental protective norms”) is much more effective than using altruism-based measuring items (i.e., "Do you have the intention to protect the environment for the benefit of the local people?"). Reasonable expectations are also an effective method. Mihalič referred to the fact that "improving the quality of the ecological environment can bring long-term benefits to the local tourism industry" [57] and be successfully to promote the individual intention of pro-environmental behavior. The results of these intentions can be explained in environmental personal norms and expectancy. Through the influence factor loadings of the final model (Fig 4), environmental personal norms were seen to be the most important influencer in stimulating the sustainable environmental protective intentions of farmers. Hence, the focus of supporting ecological environmental protection of tourist destinations should be placed at the environmental personal norm level.

The Chinese government pays increasing attention to the ecological environment of tourist destinations because local governments have gradually realized the importance of sustainable development. The total variable analysis of the valence interpretation supports the implementational possibility of a reward system for farmers in tourist destinations. De Young proposed a reward system related to environmental protection to help promote the possibility of implementing conservation behaviors [58]. This reward system for individuals requires environmental conservation participants to increase their investment of capital and time for environmental restoration, raising the question of whether tourism revenue after implementing environmental protection investment can be higher than the initial high cost of implementing the corresponding investment. However, while this approach might pose a challenge to the sustainable development of rural tourism in the future, it could still cost a certain amount of time, capital, and labor. Expectancy makes another important practical contribution to destination managers by explaining the importance of guiding personal attitudes and expectations for pro-environmental behavior intentions. Therefore, the concept bridges the gap between attitude and behavior. Moreover, in theory, these contributions make it more positive for farmers to generate intentions to protect the environment according to the rules of influence between them, for example, to help promote the pro-environmental behavior of farmers. It further emphasizes that the attitude of learning environmental protection knowledge needs wider public support and advocacy. At the same time, it is also worth noting that improving the ecological environment for farmers also benefits foreign tourists.

## Conclusion

The norm activation model was proposed by Schwartz, and it has been developed and improved; however, it has not been widely recognized in growing research fields or context changes. Since the introduction of the NAM, many studies have validated the rationality of its construction in various contexts involving the exploration of conservation behavior. By demonstrating the effective application of the model to farmers’ intentions, the current study added a new research context to the intention of pro-environmental behavior. Hence, the model successfully adds another empirical context. The theoretical implications of this study mainly lie in its accuracy in predicting environmental behavior intention, its comprehensiveness in theoretical broadening and deepening, and the research objectives of this study. In addition, this study expands the framework of the norm activation model, and it demonstrates the validity of extending the norm activation model to slightly different content, such as the pro-environmental behavior intention of farmers engaged in tourism when their environment is suffering from damage caused by tourist destinations in China due to excessive development and reception.

Therefore, the theoretical contributions of this paper with practical significance are as follows: we go beyond the existing model and expand it to a refined model based on the norm activation model and expectancy theory. The model can be specifically applied to the field of a rural tourism area with its tourism as the leading industry. This paper analyzes the changing rules of the pro-environmental intention of local farmers who are engaged in tourism from two analysis dimensions of norm activations and expectations rewards, which has not been explained by the existing literatures on pro-environmental behavior in tourist destinations. From the model fit results, pro-environmental intentions are mainly affected by environmental personal norms (the impact load coefficient is as high as 0.739) and by a certain degree of personal expectancy (the impact load coefficient is 0.173); From the discussion, the improvement of local farmers’ pro-environmental intention needs two main approaches: the cultivation of personal environmental protection norms and the guidance of producing a desired expectation for pro-environmental intention. Our theoretical framework involving six constructs and six model hypotheses has been satisfactorily supported; while H6 was not significant, and D1 was found to be a new causal relationship. Although the existing research has rarely merged the NAM and expectancy theory into a multidimensional theoretical framework, this merged model reflects the cross-effects of related factors from the two theories, and these influences may be closer to the real situation of the farmers’ pro-environmental intentions in tourist areas where the ecological environment is polluted. This research can provide a theoretical basis for government managers to improve farmers’ pro-environmental intentions and provide valuable and unique views for the sustainable development of rural tourism in specific world-famous rural tourist destinations. In addition, this research can help to further enrich the literature on rural tourism concerning pro-environmental behaviors and help rural tourism managers find better measures to improve local farmers’ pro-environmental intentions.

## Limitation and further research

In this paper, the improvement of farmers’ pro-environmental intention in the destroyed tourist destinations are based on the fitting results of the proposed research model. We propose improvement measures in two aspects: the cultivation of personal environmental protection norms and the guidance of producing a desired expectation for pro-environmental intention. Combined with my own understanding of the actual situation of the research destination, these improvements are effective for the tourist case. But as to whether these improvements are applicable to other types of tourist destinations, this question needs further research work in the future. This challenging question also points out the direction for testing the effectiveness of countermeasures for my future research on the pro-environmental behavior of tourist destinations.

## Supporting information

S1 File Investigation on farmers’ intention of pro-environmental behavior in Guangming village. (DOCX)

S1 Table Data used in structural equation analysis. (XLS)
