# Supplementary material for: Analyzing the farmers’ pro-environmental behavior intention and their rural tourism livelihood in tourist village where its ecological environment is polluted
Source: PLoS One. 2021 Mar 11;16(3):e0247407. doi: 10.1371/journal.pone.0247407 (PMC7951871; doi:10.1371/journal.pone.0247407)
Supplement: S1 File — (DOCX) [file pone.0247407.s001.docx]

**S1 File. Investigation on farmers' intention of pro-environmental behaviour in Guangming Village**

*Awareness of environmental Consequences.* Extremely disagree (1)/Extremely agree (5).

The tourism industry can cause pollution, climate change, and exhaustion of natural resources because many tourists flow into tourist destinations in a short time.

A good tourism environment is related to the sustainable livelihoods of local farmers.

Tourism can generate huge environmental impacts on local tourist attractions.

Large-scale tourists may lead to the deterioration of the ecological environment, because farmers overuse goods / energy / water in order to receive tourists.

*Ascription of environmental responsibility.* Extremely disagree (1)/Extremely agree (5).

The correct ascription of environmental responsibility helps farmers to expect the desired outcomes.

I believe that local farmers should be partially responsible for environmental problems caused by tourism.

I feel that local farmers should be responsible for actions that lead to environmental degradation in order to obtain tourism benefits.

Farmers near tourist destinations should be responsible for neglecting the proper handling of environmental issues in the development of rural tourism.

*Environmental personal norm.* Extremely disagree (1)/Extremely agree (5).

I consider that some farmers in the tourist destination are need to learn relevant environmental protection norms.

I feel obliged to choose pro-environmental activities and act in an environmentally friendly manner in order to achieve the sustainable development goals of rural tourism.

I think it is more important to be environmentally friendly and reduce the damage to the ecological environment of tourist destination during the agricultural production process.

When the country is paying more and more attention to the sustainable development of rural tourism, I should show our pro-environmental intentions to our beautiful tourist destinations.

*Pro-environmental intention*. Extremely disagree (1)/Extremely agree (5).

I would give priority to accept the guidance of agricultural experts on how to carry out agricultural activities reasonably around the tourist destination.

I would sacrifice my rest time to promote the environmental protection policy.

I would persuade others to protect the ecological environment of tourist destination.

I would spend more money to improve the tourism service facilities in order to reduce environmental pollution.

I would learn relevant environmental protective skills and knowledge.

*Valence*. Extremely disagree (1)/Extremely agree (5).

I believe that if the ecological environment of tourist attractions and their communities is good, I will enjoy the long-term development benefits brought by tourism.

I believe that carrying out pro-environmental activities in daily agricultural production or tourism services is beneficial for protecting the environment of tourist destinations.

Unpolluted and clean tourist destinations are essential to my sustainable income from tourism.

*Expectancy*. Extremely disagree (1)/Extremely agree (5).

The more your expectations, the more positive behavioural intentions to achieve the tasks of the environmental protection.

I believe improve the quality of ecological environment can bring long-term benefits to the local tourism industry.

My pro-environment behaviours can prevent environmental degradation.

I believe that environmental protection does not require more time and capital costs.
